# Endotoxin removal by hemoperfusion in septic shock

**DOI:** 10.1186/cc10376

**Published:** 2011-10-27

**Authors:** B Adamik, J Smiechowicz, S Zielinski, A Kübler

**Affiliations:** 1 Department of Anesthesia and Intensive Care, Wroclaw University of Medicine, Wroclaw, Poland

## Introduction

Many symptoms of septic shock are due to the presence of endotoxin in the bloodstream. The biological activity of endotoxins is associated with lipopolysaccharide (LPS). LPS induces systemic inflammatory response and a high level of endotoxin in blood is associated with worse clinical outcome. Reduction of the level of circulating endotoxins with hemoperfusion through the filter with high affinity for LPS could potentially interrupt the biological cascade of sepsis. The aim of the study was to evaluate the efficiency of extracorporeal endotoxin elimination in patients with Gram-negative septic shock.

## Methods

The study was conducted at the Department of Anesthesiology and Intensive Therapy, Wroclaw Medical University, Poland. Patients with septic shock, documented or suspected Gram-negative infection, and with high endotoxin activity (EA >0.6 units) were eligible for the study. The endotoxin activity in blood was measured with chemiluminescent activity assay. Based on the enrolment criteria and EA level, patients were assigned to the conventional treatment group (Group 1) or the conventional plus hemoperfusion therapy with LPS adsorber (Alteco Medical AB, Lund, Sweden) group (Group 2). Hemoperfusion was performed for 2 hours with blood flow maintained at 150 ml/minute.

## Results

Seventeen patients with low EA (0.42 ± 0.14, Group 1) and 12 patients with high EA (0.76 ± 0.13, Group 2) (*P *< 0.05) were included. There were no significant differences between Group 1 and 2 regarding age (63 ± 2 and 61 ± 21), APACHE II score (22.7 ± 8.6 and 24.5 ± 7.2), SOFA score (9.8 ± 3.0 and 11.3 ± 4.1), mean arterial pressure (MAP, 66.2 ± 8.1 mmHg and 71.5 ± 7.3 mmHg), and PaO_2_/FiO_2 _(255 ± 59 and 216 ± 105) at entry to the study. In the hemoperfusion group, nine patients had Gram-negative and three had Gram-positive infection; seven patients survived to the 28-day follow-up. High endotoxin activity at baseline decreased significantly 24 hours after hemoperfusion to 0.5 ± 0.1 (*P *< 0.01) in those who survived, but remained high (0.7 ± 0.1) in nonsurvivors. At 24 hours after hemoperfusion, MAP significantly increased (78.8 ± 20.8 to 89.2 ± 19.8 mm Hg, *P *< 0.05) and vasopressor requirements decreased in survivors but not in those who died (MAP, 64.2 ± 9.6 to 71.8 ± 15.3 mm Hg, *P *= nonsignificant).

## Conclusion

Hemoperfusion with LPS adsorber added to standard treatment improved the hemodynamic status of patients with septic shock. The chemiluminescence assay for measurement of LPS activity was a valuable diagnostic tool for rapid detection of endotoxemia.

